# Periaortic adipose radiomics texture features associated with increased coronary calcium score—first results on a photon-counting-CT

**DOI:** 10.1186/s12880-023-01058-7

**Published:** 2023-07-26

**Authors:** Peter Mundt, Hishan Tharmaseelan, Alexander Hertel, Lukas T. Rotkopf, Dominik Nörenberg, Philipp Riffel, Stefan O. Schoenberg, Matthias F. Froelich, Isabelle Ayx

**Affiliations:** 1 Department of Radiology and Nuclear Medicine, University Medical Centre Mannheim, Heidelberg University, Theodor-Kutzer-Ufer 1-3, 68167 Mannheim, Germany; 2 Department of Radiology, German Cancer Research Centre, Im Neuenheimer Feld 280, 69120 Heidelberg, Germany

**Keywords:** Photon-counting computed tomography, Coronary artery calcium score, Radiomics, Texture analysis

## Abstract

**Background:**

Cardiovascular diseases remain the world’s primary cause of death. The identification and treatment of patients at risk of cardiovascular events thus are as important as ever. Adipose tissue is a classic risk factor for cardiovascular diseases, has been linked to systemic inflammation, and is suspected to contribute to vascular calcification. To further investigate this issue, the use of texture analysis of adipose tissue using radiomics features could prove a feasible option.

**Methods:**

In this retrospective single-center study, 55 patients (mean age 56, 34 male, 21 female) were scanned on a first-generation photon-counting CT. On axial unenhanced images, periaortic adipose tissue surrounding the thoracic descending aorta was segmented manually. For feature extraction, patients were divided into three groups, depending on coronary artery calcification (Agatston Score 0, Agatston Score 1–99, Agatston Score ≥ 100). 106 features were extracted using pyradiomics. R statistics was used for statistical analysis, calculating mean and standard deviation with Pearson correlation coefficient for feature correlation. Random Forest classification was carried out for feature selection and Boxplots and heatmaps were used for visualization. Additionally, monovariable logistic regression predicting an Agatston Score > 0 was performed, selected features were tested for multicollinearity and a 10-fold cross-validation investigated the stability of the leading feature.

**Results:**

Two higher-order radiomics features, namely “glcm_ClusterProminence” and “glcm_ClusterTendency” were found to differ between patients without coronary artery calcification and those with coronary artery calcification (Agatston Score ≥ 100) through Random Forest classification. As the leading differentiating feature “glcm_ClusterProminence” was identified.

**Conclusion:**

Changes in periaortic adipose tissue texture seem to correlate with coronary artery calcium score, supporting a possible influence of inflammatory or fibrotic activity in perivascular adipose tissue. Radiomics features may potentially aid as corresponding biomarkers in the future.

## Background

As cardiovascular diseases (CVD) are still the world's leading cause of death [1], identifying and treating patients at risk of ischemic events remains a vital task of modern medicine. Coronary artery calcification (CAC) is an important risk factor for CVD, which is why the coronary artery calcium score (CACS) has proven to be a reliable marker for risk stratification of corresponding patients [2]. In daily practice, the Agatston Score is used for estimating the extent of CAC differentiated to main coronary arteries [3]. Depending on the severity of calcifications and hence the Agatston Score, the probability for the development of an obstructive coronary artery disease event can be estimated, outlining a cut-off value of 100 as a reference point for the occurrence of most coronary events such as myocardial infarction and death [4].

Another important influence are various adipose tissues, which have traditionally been linked to multiple other cardiovascular risk factors [5]. In several studies, these were found to contribute to systemic inflammation [6] and were correlated to atherosclerosis [6–8]. Especially intrathoracic, periaortic (PAAT), and epicardial adipose tissue (EAT) were linked to vascular calcification [6–8], suggesting possible local toxic effects on the vasculature [8].

In most studies, adipose tissue was examined mainly in terms of volume [5–9] and density [10–12], hence there is little data on tissue characteristics, especially from PAAT and EAT. A possible way to address this issue could be the use of texture analysis.

Radiomics is a rapidly evolving field of medical imaging, analyzing pixel-based information within the image beyond the natural limitations of the examiner's eye. These so-called features are quantitative metrics regarding various characteristics of the region/volume of interest (ROI/VOI) such as shape and texture and may, either alone or in correlation with further patient data, help to solve diagnostic challenges or even serve as prognostic biomarkers [13]. Radiomics features are mineable, meaning with a sufficiently large database, new features may be discovered as potential markers or patterns of certain lesions or diseases [14].

While radiomics is already well established in oncology research [13, 15], the use of radiomics in cardiac imaging, and therefore its application regarding EAT and PAAT is still in its early stages [16]. Thus, the amount of data on radiomics features in cardiovascular imaging is still relatively small. However, initial studies on the use of radiomics in this area suggest possible benefits [17].

For higher accuracy in radiomics features, a silicon-based photon-counting computed tomography (PCCT) offers an alternative to conventional energy-integrating computed tomography (EICT) due to improved spatial resolution [18]. PCCT is a ground-breaking technology that has been shown to improve the quality of computed tomography (CT) imaging and allows a higher contrast-to-noise ratio and a higher spatial resolution [19]. This novel technology has the chance to overcome the limitations of texture analysis by increased spatial resolution and may pave the way for a sufficient analysis of PAAT.

Hence, this study aims to investigate a possible correlation between the extent of CAC and PAAT texture features on PCCT images and possibly find a potential biomarker for the diffuse inflammatory reaction of PAAT leading to coronary artery sclerosis.

## Methods

### Study design

For this retrospective single-center study patients with suspected or known coronary artery disease (CAD) and clinically indicated electrocardiography (ECG)-gated cardiac CT were enrolled between December 2021 and March 2022. Patients were excluded in case of severe image artifacts, i.e. motion artifacts (*n* = 3), or in case of previous cardiac stent implantation (*n* = 5). The patient population was screened for metal artifacts arising from i.e. dorsal spinal fusion (*n* = 0) or pacemaker device (*n* = 0) and for potential mass or visible tissue inhomogeneities affecting the PAAT (*n* = 0). The study had an institutional review board and local ethics committee approval (ID 2021–659, Ethikkomission II, Heidelberg University).

### Patient collective and cardiac CT imaging protocol

Based on inclusion and exclusion criteria, a total of 55 patients (34 male, 21 female, mean age 56 years, range: 20–80 years) were enrolled in this study. All included 55 patients were scanned on a first-generation whole-body dual-source PCCT system (NAEOTOM Alpha; Siemens Healthcare GmbH, Forchheim, Germany). For examination, a prospective ECG-gated sequential mode with a tube voltage of 120 kV and automatic dose modulation with a CARE keV BQ setting of 64 was used. Effective gantry rotation time was 0.25 s. In correlation to heart rate and in absence of contraindications, patients received 5–10 mg of metoprolol intravenously to lower heart rates sufficiently. Additionally, patients received, in absence of contraindication, 0.4–0.8 mg nitroglycerin. For evaluation of CAC, all patients underwent a non-contrast-enhanced cardiac CT. This scan was followed by a contrast-enhanced scan of the coronary arteries using 80 ml of iodine contrast (Imeron 400, Bracco Imaging Deutschland GmbH, Konstanz, Germany) and 20 ml saline chaser (NaCl 0,9%) with a weight-based flow rate of 4–5 ml/sec via antecubital venous access.

### Cardiac CT imaging analysis

**Fig. 1:**
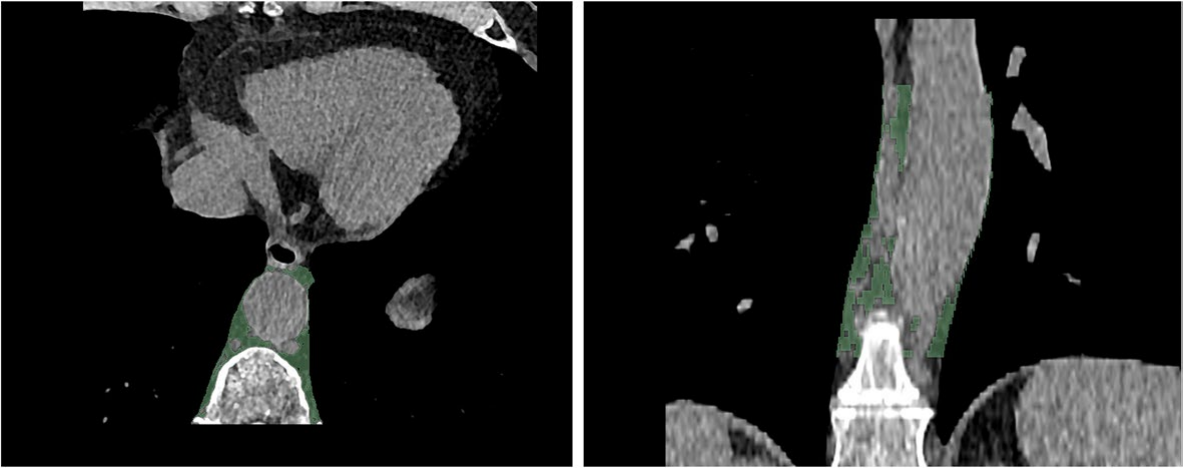
Segmentation of the periaortic thoracic adipose tissue (PAAT) was performed on axial view with a slice thickness of 2 mm (green area, left side) building a segmentation tube around the descending aorta (green area, coronal view, right side)

Axial non-enhanced images were reconstructed with a slice thickness of 2 mm (increment 2 mm) using a soft vascular kernel (Bv40). This data was anonymized, exported, and stored in digital imaging and communication in medicine (DICOM) file format. DICOM files were converted to Neuroimaging Informatics Technology Initiative (NIFTI) file format for utilization with a dedicated segmentation tool (3D Slicer, Version 4.11) [20]. PAAT surrounding the descending thoracic aorta was segmented manually with a threshold of -195/-45 Hounsfield units (HU) [21] by a medical student and reviewed by a board-certified radiologist with 9 years of clinical experience in cardiovascular imaging. The segmentation included a rectangle of adipose tissue defined by the esophagus as the ventral border and the costovertebral angle as the dorsal border as recommended by Turkem et al. [22], shown in Fig. 1. The cranial border for segmentation was set at the pulmonary trunk and extended over 8 cm downward. Additionally, signal-to-noise ratio (SNR) was calculated for EAT by defining a specific ROI next to the right coronary artery (RCA). Mean HU was divided through standard deviation (SD) within the ROI.

Calcium scoring was performed by a radiologist with 9 years of experience in cardiac imaging on axial non-enhanced scans with 3 mm slice thickness and a quantitative Qr36 kernel using dedicated software (syngo.via, Siemens Healthcare GmbH, Forchheim, Germany) for the calculation of the Agatston Score. Afterward, the study population was split up into three different groups depending on the severity of CAC: patients with no sign of CAC (Agatston Score 0), patients with Agatston Scores between 1—99, and patients with Agatston Scores ≥ 100. The cut-off value of 100 was chosen in line with the literature, as this is associated with a significant increase in the development of coronary events [4, 23].

### Radiomics feature extraction and statistical analysis

Features for the PAAT regions of interest were further extracted using a dedicated imaging biomarker standardization initiative definition (IBSI)-based python package (pyradiomics, version 3.0.1.) [24]. For each patient enrolled in this study first-order features and second-order features, namely grey level co-occurrence matrix (glcm), grey level dependence matrix (gldm), grey level size zone matrix (glszm), grey level run length matrix (glrlm), and neighbouring grey tone difference matrix (ngtdm) were extracted.

All statistical analyses were performed in R [25] and RStudio (version 1.3.1093, Boston, MA, USA) [26]. For all quantitative parameters mean and SD were calculated, categorical variables were summarized as percentages. Normalization of all radiomics features was achieved using z-score:

$$\mathrm{z}=((\mathrm{X}-\upmu ))/\upsigma ,$$

with µ being the mean and σ the feature SD. Pearson correlation coefficients were used for the correlation of feature calculation. Features were visualized in boxplots and heatmaps using the ComplexHeatmap Package in R. Hierarchical clustering was performed within each Agatston Score Group. A permutation-based Random Forest (RF) classifier was applied with the Boruta package for R for feature selection by the calculation of feature importance. For group comparisons, student’s t-test was performed, and for multiple-group comparisons, ANOVA was performed. Additionally, monovariable logistic regression predicting an Agatston Score > 0 was performed. Furthermore, relevant features were investigated for multicollinearity, and a 10-fold cross-validation was performed for the leading feature.

## Results

### Patient and image characteristics

The patient’s characteristics depending on the severity of CAC are summarized in Table 1.

**Table 1:** Patient collective overview. Mean and (SD) given for continuous variables

	**Overall Patients**	**Agatston 0**	**Agatston 1–99**	**Agatston ≥ 100**	***p*-value**
**n**	55	23	19	13	
**Age (mean(SD))**	56.17 (13.24)	47.30 (13.98)	61.79 (10.21)	60.46 (8.96)	< 0.001
**Sex (%)**					
Male	34 (61.81%)	13 (56.5%)	10 (52.6%)	11 (84.6%)	0.148
Female	21 (38.18%)	10 (43.5%)	9 (47.4%)	2 (15.4%)	
**Agatston Score (mean(SD))**	169.87 (457.66)	0 (0)	24.56 (20.78)	676.12 (797.49)	< 0.001

For all patients, SNR was calculated as described in the material and method section, mean SNR for the EAT of our patient group was 3.82.

For all patients, the mean HU value of the whole segmentation was determined (Agatston Score 0: -97,97 HU, Agatston Score 1–99: -101,69 HU, Agatston Score ≥ 100: -100,38 HU). Additionally, the volume of the PAAT in all three patient populations was quantified as outlined in Table 2. Clinical data were retrospectively evaluated, hence only in 25 out of 55 patients cardiovascular risk factors could be determined (Table 2).

**Table 2:** Conventional and clinical patients’ parameters. Mean and (SD) given for continuous variables

	**Overall Patients**	**Agatston 0**	**Agatston 1–99**	**Agatston ≥ 100**	***p*-value**
**n**	**55**	**23**	**19**	**13**	
Agatston Score (mean (SD))	169.87 (457.66)	0 (0)	24.56 (20.78)	676.12 (797.49)	< 0.001
PAAT mean density (in HU Value (SD))	-99.83 (26.13)	-97.97 (5.54)	-101.69 (5.20)	-100.38 (6.06)	0.098
PAAT mean volume (in mm^3^ (SD))	78968.48 (45,572.69)	64832.28 (47194.59)	89429.63 (45965.33)	87601.91 (38535.90)	0.031
**Patients with known cardiovascular risk factors**	**25**	**8**	**10**	**7**	< 0.001
Hypertension (n)	19	5	9	5	< 0.001
Diabetes (n)	1	1	0	0	-
Dyslipidemia (n)	4	3	1	0	0.83
Nicotine abuse (n)	6	1	2	2	0.117

### Cluster analysis

**Fig. 2:**
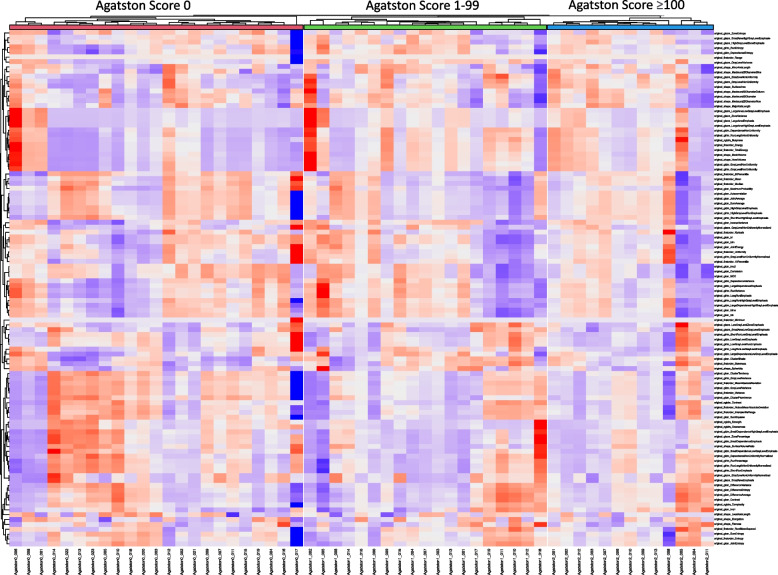
Unsupervised cluster heatmap of aggregated radiomics features of periaortic adipose tissue (PAAT)

Hierarchical clustering of the radiomics features extracted from the PAAT of every patient was performed after standardization, as well as clustering within each Agatston Score group. These results were demonstrated in a heatmap (Fig. 2).

### Feature selection

**Fig. 3:**
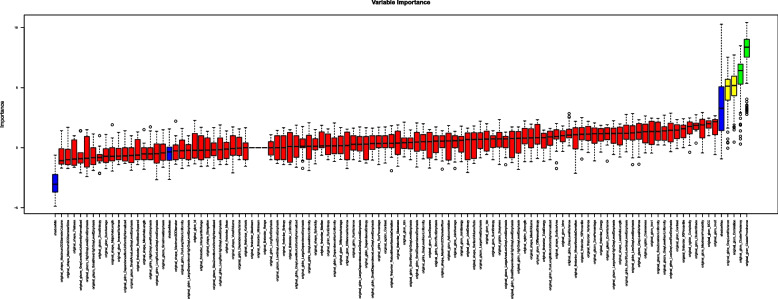
Random Forest (RF) feature selection in the 36 patients of the validation set (differentiating features marked in green)

Important features for differentiation based on the PAAT texture were selected by Boruta feature selection: RF-based feature selection was performed on the Agatston Score groups Agatston 0 and Agatston ≥ 100. In the process, out of 106 features, two second-order features, namely “original_glcm_ClusterTendency” and “original_glcm_ClusterProminence”, were identified as features associated with a difference in Agatston Score (Fig. 3).

### Internal validation

**Fig. 4:**
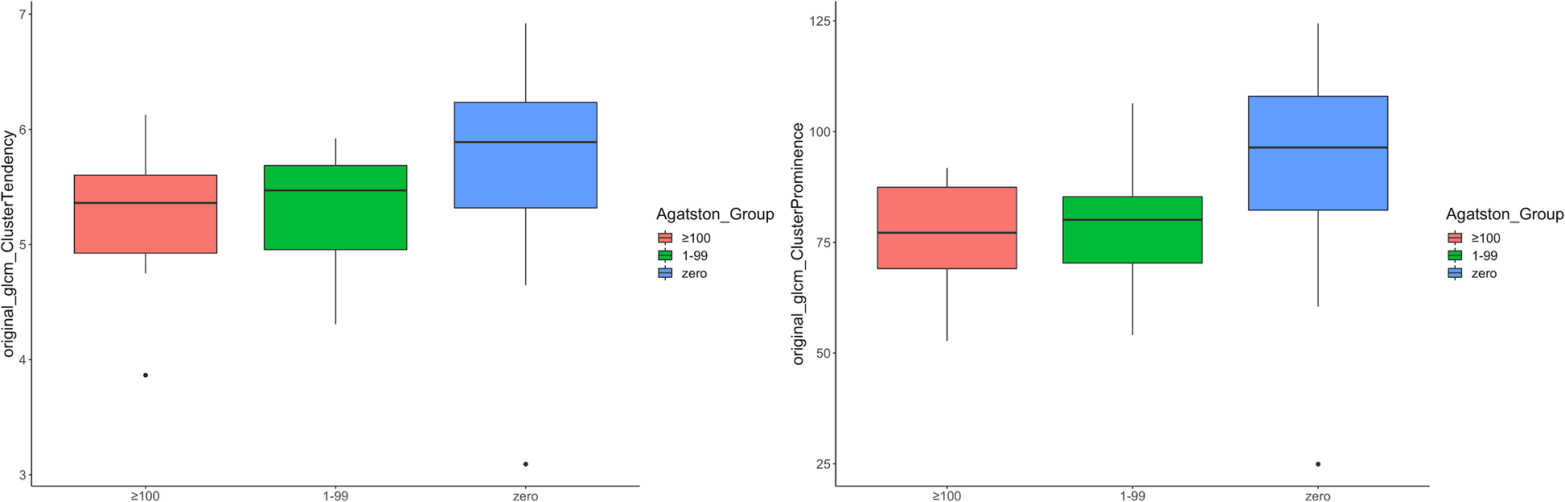
Distribution of “original_glcm_ClusterProminance” and “original_glcm_ClusterTendency” features within the dataset (orange = Agatston Score ≥ 100, green = Agatston Score 1–99, blue = Agatston Score 0)

For internal validation, the aforementioned features were additionally investigated in the group of Agatston Score 1–99. The corresponding radiomics features are shown as boxplots in Fig. 4 and summarized in Table 3.

**Table 3:** Differentiating higher order radiomics features. Mean and (SD) given for continuous variables

	**Agatston 0**	**Agatston 1–99**	**Agatston ≥ 100**	***p*-value**
**original_glcm_ClusterProminence**	92.77(22.86)	79.23(13.14)	77.05 (0.53)	0.019
**original_glcm_ClusterTendency**	5.72 (0.84)	5.33 (0.46)	5.24 (0.53)	0.078

Supporting the expected change of texture in PAAT with an increasing Agatston Score, the Agatston Score 1–99 group was placed in between the two other groups, resulting in the respective mean values (Agatston Score 0/1–99/ ≥ 100) of 92.77/79.23/77.05 for “original_glcm_ClusterProminence” (*p* = 0.019) and 5.72/5.33/5.24 for “original_glcm_ClusterTendency” (*p* = 0.078).

#### Internal cross-validation and assessment for multicollinearity

To investigate the association of the variables “original_glcm_ClusterProminence” and “original_glcm_ClusterTendency” with an increased Agatston Score, additional monovariable, logistic regressions predicting an Agatston Score of > 0 were performed. The corresponding results are shown in Table 4.

**Table 4:** Logistic regression for prediction of Agatston Score > 0

	**Model 1**		**Model 2**		**Combined Model**	
	Predictor	*p*	Predictor	*p*	Predictor	*p*
Intercept	4.39025	0.0065	6.0384	0.0263	1.55465	0.66
original_glcm_ClusterProminence	-0.04741	0.0097			-0.08217	0.0656
original_glcm_ClusterTendency			-1.0339	0.0332	1.05091	0.3846

**Fig. 5:**
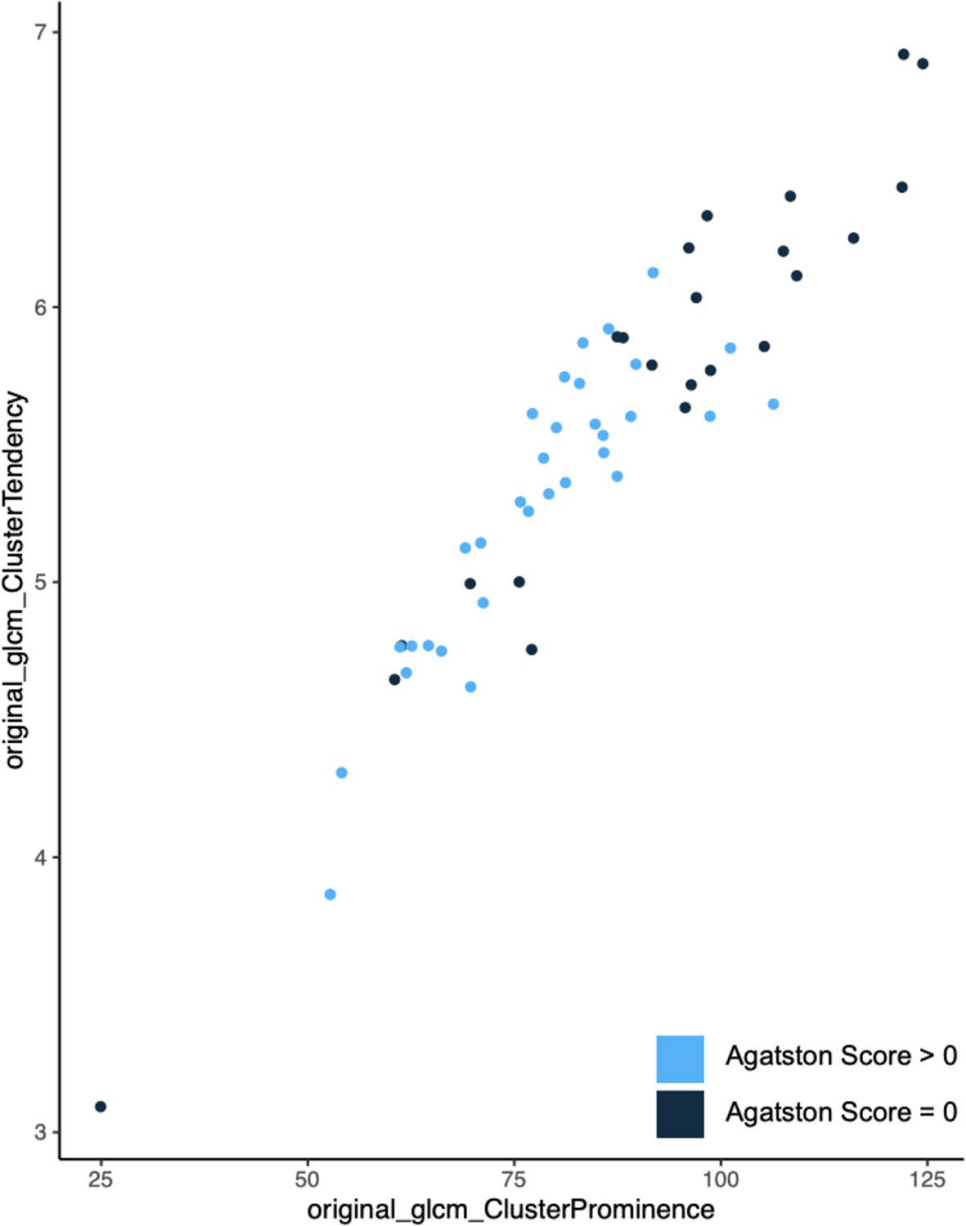
Association of variables “original_glcm_ClusterProminence” and “original_glcm_ClusterTendency”

To assess for multicollinearity, Variance Inflation Factor (VIF) was calculated and resulted in a value of 7.9269, indicating multicollinearity. The association of both variables is visualized in Fig. 5.

As a result, the variable “original_glcm_ClusterProminence” was selected for the final model. A ten-fold cross-validation of the logistic regression was performed to investigate its stability. This resulted in a Root Mean Squared Error (RMSE) of 0.4528, a R-squared value of 0.3677, and a mean absolute error of 0.4220.

## Discussion

This study is the first to investigate the possible connection between CAC and PAAT texture changes using radiomics features on PCCT. Two features, “original_glcm_ClusterTendency” and “original_glcm_ClusterProminence”, were found to differ between different levels of CAC (Agatston Score 0/1–99/ ≥ 100). Due to the multicollinearity of the two features, “original_glcm_ClusterProminence” was selected as the leading feature, offering a potential radiomics signature for inflammatory or fibrotic changes in PAAT. “ClusterProminence” can be used as a marker for skewness and asymmetry of gray level co-occurrence matrix features. In analyses, a higher value implies more asymmetry about the mean [27]. Our study showed significantly higher values of “ClusterProminence” in the patient group without CAC (Agatston Score 0), indicating a higher asymmetry of grey values in PAAT. A possible explanation could be that diffuse fibrotic or inflammatory changes lead to a more homogenous structure, although this cannot be proven through this study. However, as adipose tissue and its inflammatory reactions have been linked to vascular calcification [6] and possible local effects [8], these preliminary findings may support the possible correlation between PAAT texture and the calcification of coronary arteries.

The correlation between adipose tissue density and cardiovascular risk factors or atherosclerosis is disputed. While there are findings on lower CT attenuation in visceral and subcutaneous fat correlating with lower risks of atherosclerosis [11], there are also findings suggesting lower density in abdominal fat to correlate with greater cardiometabolic risks [28]. The data of this study cannot follow either of these findings, as we were unable to find a significant difference in PAAT density between the three groups.

In 2010 Lehman et al. investigated a possible connection between PAAT, metabolic risk factors, and vascular calcification by quantifying PAAT and found it to be associated with CAC as well as aortic calcification [7]. Efe et al. investigated the relationship between PAAT and pericardial adipose tissue with CAD in 2014, based on PAAT and pericardial adipose tissue volume measurements. Patients were divided into two groups for PAAT (PAAT < / ≥ 24.3 cm^3^) and pericardial adipose tissue (< / ≥ 157.7cm^3^) respectively. In both groupings, the groups with higher PAAT/ pericardial adipose tissue volume had a significantly higher prevalence of CAD. [29]. Coming to a similar conclusion, Zhu et al. investigated the association of PAAT and visceral adipose tissue with coronary artery atherosclerosis in 2021, using a volume-based approach. They found PAAT volume to be significantly associated with CAD and coronary artery atherosclerosis [30]. In line with these results, the PAAT volume in this study's population did also differ significantly (*p* = 0.031) between the group with an Agatston Score of 0 and the groups with an Agatston Score ≥ 1. However, there was no significant difference in PAAT volume between the two groups with Agatston Score 1–99 (mean 89429.63 mm^3^) and Agatston Score ≥ 100 (mean 87601.91 mm^3^) and hence no linear correlation between CAC severity and PAAT volume could be found. The feature "original_glcm_ClusterProminence" did show a significant difference between all three groups, declining with increasing Agatston Score. This may imply that radiomics features could potentially aid in differentiating between the severities of CAC when a volume-based approach reaches its limitations.

In 2011 Yun et al. found adipose tissue to strongly correlate with systemic inflammation and cardiovascular risk factors when investigating a connection between pericardial fat, thoracic PAAT, and cardiovascular risk factors, as well as their value in terms of CAC. Additionally, they found pericardial fat to exert a role in CAC [6]. This supports the findings of Rosito et al. in 2008 when they found intrathoracic and pericardial fat to be associated with vascular calcification [8].

The possible connection between PAAT and vascular calcification was further supported by the findings of Tharmaseelan et al. in 2022. They investigated the correlation between abdominal PAAT and local aortic calcification by dividing 30 patients into two groups (aortic calcification present/absent) and extracting radiomics features of the abdominal PAAT. They found several abdominal PAAT radiomics texture features to be significantly associated with local aortic calcification [31], even though the significant features in their study differ from our results. A possible explanation could be that in contrast to investigating abdominal PAAT depending on local aortic calcifications, our study goes one step further and outlines a possible influence of thoracic PAAT not on local aortic calcifications but on CAC, indicating a possible more diffuse and not only local inflammatory or fibrotic process.

The results of this study must be interpreted in consideration of the following limitations. The presented study is retrospective and was performed at a single center with a limited number of patients, due to strict selection criteria and the limited number of patients who were scanned with the newly established PCCT. However, the use of a PCCT led to increased image quality and possibly more precise texture analysis. Furthermore, one severe limitation is that only insufficient clinical data were available for this study, as many patients were scanned in an outpatient setting as well as due to the retrospective nature of this study. This has to be addressed in further studies in the future. The cardiovascular risk factor “hypertension” differed significantly between the three patient groups, as did the general distribution of recorded cardiovascular risk factors, resulting in potentially contributing confounders. However, this was present mostly in the Agatston Score group 1–99 with a decrease in the patient group Agatston Score ≥ 100. This does not reflect the statistical analysis of the radiomics features, as the features present a straight falling intensity with rising CAC. Moreover, the gender distribution within the group of Agatston Score ≥ 100 is uneven, with 86% male patients, as well as the age distribution between the different Agatston Score groups, so a more balanced age and gender distribution will be needed in further investigations, preferring a prospective multicenter approach in the future with a larger patient population with sufficient clinical data. Lastly, as pointed out by Ayx et al., these results may not be fully comparable to any similar observations on EICT scanners, as their data suggest differences in radiomics feature values, especially in higher-order features such as the aforementioned ones, between EICT and PCCT scanners [32]. An additional study comparing PAAT in patients scanned on EICT and PCCT must follow in the future to address these severe limitations. Furthermore, radiomics analysis of PAAT should be performed using different reconstruction algorithms to address the limited reproducibility in our preliminary study.

## Conclusion

In conclusion, this study is the first to investigate and outline a possible correlation between PAAT texture and coronary artery sclerosis. This may allow the hypothesis of possible texture changes through inflammatory or fibrotic processes in perivascular adipose tissue influencing the process of arteriosclerosis, as well as suggesting that radiomics features may potentially serve as biomarkers for such respective changes.

## Data Availability

The dataset used and/or analyzed during the current study are available from the corresponding author on reasonable request.

## Abbreviations

CAC: Coronary artery calcification
CACS: Coronary artery calcium score
CAD: Coronary artery disease
CT: Computed tomography
CVD: Cardiovascular disease
DICOM: Digital Imaging and Communication in Medicine
EAT: Epicardial adipose tissue
ECG: Electrocardiography
EICT: Energy-Integrating computed tomography
GLCM: Grey Level Co-occurrence Matrix
GLDM: Grey Level Dependence Matrix
GLRLM: Grey Level Run Length Matrix
GLSZM: Grey Level Size Zone Matrix
HU: Hounsfield units
IBSI: Imaging biomarker standardization initiative definition
NGTDM: Neighbouring Grey Tone Difference Matrix
NIFTI: Neuroimaging Informatics Technology Initiative
PAAT: Periaortic adipose tissue
PCCT: Photon-Counting computed tomography
RCA: Right coronary artery
RF: Random Forest
RMSE: Root Mean Squared Error
ROI: Region of Interest
SD: Standard deviation
SNR: Signal-to-noise Ratio
VOI: Volume of Interest
VIF: Variance Inflation Factor
